# Commentary: HNRNPLL, a newly identified colorectal cancer metastasis suppressor, modulates alternative splicing of *CD44* during epithelial-mesenchymal transition

**DOI:** 10.3389/fcell.2017.00091

**Published:** 2017-10-16

**Authors:** Somesh Baranwal

**Affiliations:** Center for Biochemistry and Microbial Science, School of Basic and Applied Science, Central University of Punjab, Bathinda, India

**Keywords:** metastasis, shRNA, cell adhesion, mouse model, alternative splicing

Metastasis, the spread of a tumor from its primary site to a distant organ, is the leading cause of cancer morbidity and mortality (Lambert et al., 2017). Metastasis Suppressors (MS) and Metastasis Inducers (MI) genes are critical mediators of this process. By definition, MS gene inhibits metastasis without affecting *in-vitro* cell proliferation and *in-vivo* primary tumor growth (Smith and Theodorescu, 2009). To date, more than 40 metastasis suppressor genes have been reported in colorectal cancer (Zhao et al., 2015); restoration of MS gene and its activity is an attractive strategy to prevent metastasis and disease-free survival of cancer patients.

Aberrant splicing generates diversity in human proteome and emerges as a key mediator in the differentiation and epithelial-mesenchymal transition (EMT) (Biamonti et al., 2012). Differential splicing of CD44 imparts an instrumental role in metastasis and drug resistance in several cancers. CD44*v*6, a marker for cancer stem cells, has been shown to promote cancer metastasis and drive rewiring (reprogramming) of Cancer Stem Cell (CSC) in colon cancer (Todaro et al., 2014).

Heterogeneous nuclear ribonucleoprotein L-like (HNRNPLL), located on the chromosome 2p22.1, is a RNA-binding protein that functions as a regulator of alternative splicing for multiple mRNAs target. Genome-wide screening using shRNA library in the orthotropic and lung metastasis mouse model has identified several MS genes in breast and colon cancer (Gumireddy et al., 2009; Duquet et al., 2014). Using a genome-wide silencing approach in non-metastatic CMT93 mouse cell line expressing Venus fluorescent protein in a syngeneic mouse model, Sakuma and colleagues have delineated the inverse relationship between HNRNPLL and CD44*v*6 expression in the metastasis of colon cancer (Sakuma et al., 2017).

shRNA mediated silencing of HNRNPLL inhibits *in-vitro* cell proliferation and enhances matrigel invasion to promote metastasis in colon cancer cells. Further, knocking down HNRNPLL inhibits *in-vivo* primary tumor growth, and lung metastasis of colon cancer which is reverted by shRNA resistant HNRNPLL plasmid. RNA immunoprecipitation (RIP) analysis in HNRNPLL silencing SW480 cells increases expression of CD44 exon *v*3-10 while exogenous expression of FLAG-tagged HNRNPLL-infected cells shows a significant increase in the CD44*v*6 expression. Further, Western blot analysis revealed the significantly higher expression of CD44*v*6 in the HNRNPLL-depleting CMT93 cells. Interestingly, pre-incubation with 2F10 and 9A4 neutralizing antibodies for CD44*v*6 reduced the matrigel invasion in control and HNRNPLL-knocked down cells confirming its function in promoting invasion of colon cancer cells. Moreover, knocking down HNRNPLL promotes CD44*v*6 mediated HGF induced c-Met phosphorylation to promotes invasion in colon cancer cells.

Epithelial-mesenchymal transition emerges as a driving force for the tumor metastasis, drug resistance, and cancer stem cell generation (Shibue and Weinberg, 2017). Mechanistically, HNRNPLL expression was significantly reduced both at the mRNA and protein level in the HGF/FGF-induced *in-vitro* epithelial-mesenchymal transition in SW480 colon cells which is recovered by mesenchymal-epithelial transition (MET). Moreover, immunostaining analysis of tumor samples shows the substantially decreased expression of HNRNPLL at the invasive front with E-cadherin suggesting its function in regulating early event in metastasis. Further, Induction of EMT specifically downregulated expression of HNRNPLL without much change in closely related splicing factors such as Heterogeneous Nuclear Ribonucleoprotein M (HNRNPM), Heterogeneous Nuclear Ribonucleoprotein L (HNRNPL) and epithelial splicing regulatory protein 1 (ESRP1). In summary, their data provide the critical role of HNRNPLL in regulating tumor cell invasion and epithelial-mesenchymal transition to regulate metastasis of colon cancer cells (Figure 1).

**Figure 1:**
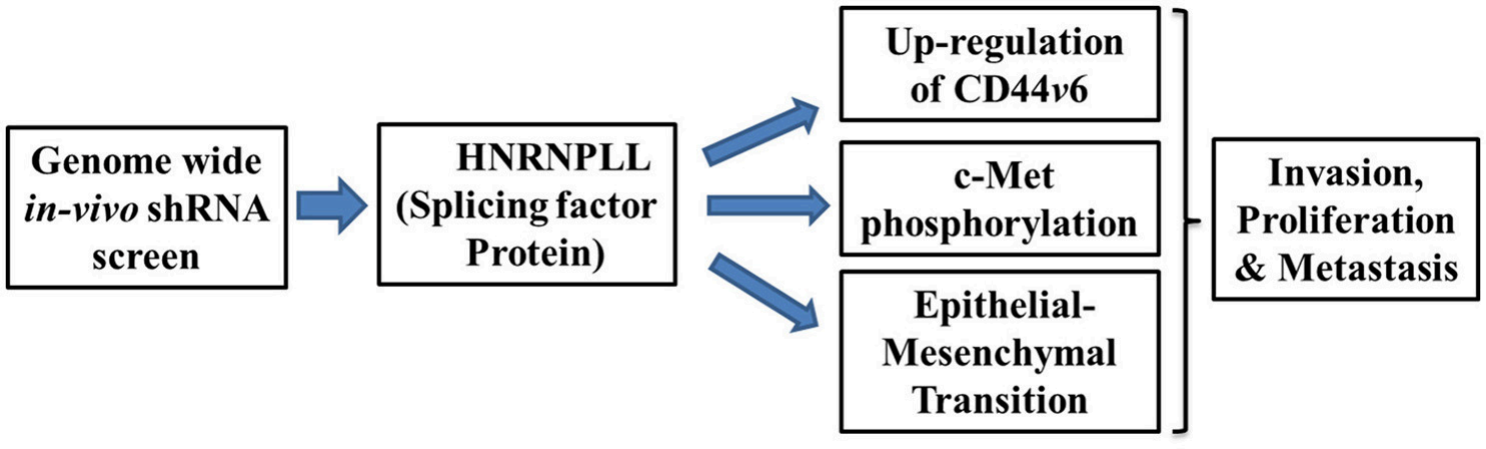
Schematic illustration showing mechanisms of metastatic suppressor function of HNRNPLL. Silencing of HNRNPLL promotes alternate splicing of CD44*v*6 by exon inclusion, c-Met phosphorylation and epithelial-mesenchymal transition to regulates invasion, proliferation and metastasis in colon cancer.

HNRNPLL expressed ubiquitously with strong nuclear and cytoplasmic expression with at least 9 predicted transcript variant of molecular weight ranging from 16.1 to 60.1 kDa (Uhlen et al., 2015). HNRNPLL predicted to undergo post-translational modification such as ubiquitination and phosphorylation^1^. Future studies will elucidate the transcript-specific roles of HNRNPLL in the regulation of metastasis, and exact function of post-translational modification in the stability and function of HNRNPLL.

Despite these, the exact mechanism of how and when HNRNPLL expression is lost or suppressed, during colon cancer progression remains to be characterized. Further, given its critical importance in EMT transition, it will be plausible to see whether HNRNPLL regulates CSC growth, self-renewal, and drug resistance in colon cancer. Moreover, how expression level of HNRNPLL in a large cohort will serve as a prognostic marker and whether HNRNPLL expression can be used as tools for predicting drug treatment for colon cancer metastasis needs to be determined. In summary, future studies will unleash the detailed molecular mechanisms of HNRNPLL on the regulation of cancer progression and provide a way for targeting the early events in the colorectal cancer metastasis.

## Author contributions

The author confirms being the sole contributor of this work and approved it for publication.

### Conflict of interest statement

The author declares that the research was conducted in the absence of any commercial or financial relationships that could be construed as a potential conflict of interest.

## Footnotes

^1^http://www.uniprot.org/uniprot/Q8WVV9.
